# Bioaccumulation and toxicity of imatinib, cyclophosphamide and its transformation product carboxyphosphamide in the freshwater mussel *Elliptio complanata*

**DOI:** 10.1007/s10646-025-02976-8

**Published:** 2025-10-10

**Authors:** E Eysseric, L Vlassopoulos, C André, F Gagné, C. Gagnon

**Affiliations:** Environment and Climate Change Canada, Aquatic Contaminant Research Divison, 105 McGill, Montreal, Qc H2Y 2E7 Canada

**Keywords:** Pharmaceuticals, Anticancer drugs, Sublethal toxicity, Uptake, Surface waters

## Abstract

Pharmaceutical compounds primarily enter the environment through wastewater treatment plants, where they are not completely removed. Antineoplastic drugs, such as cyclophosphamide and imatinib, are particularly concerning due to their cytotoxic effects at low concentrations. The purpose of this study was to determine the bioavailability and toxicity of these 2 drugs to freshwater mussels, known to thrive in urban areas. Freshwater mussels *Elliptio complanata* were exposed to increasing concentrations (4, 20, and 100 µgL^− 1^) of cyclophosphamide and imatinib for 96 h at 15 °C. Although a low bioaccumulation factor of 0.27 was measured for cyclophosphamide, its level in tissues was correlated with lipid peroxidation in gonads and gonadosomatic index (GSI), which could suggest that it is involved in oxidative stress and increased GSI. The metabolite carboxyphosphamide was never detected in mussel tissue. Imatinib was more bioavailable with a measured bioaccumulation factor of 2.3. Tissue levels of imatinib were correlated with DNA damage (DNAd) in both the digestive gland and gonads while decreased acetylcholine esterase activity was observed, suggesting that it is involved in decreased neural activity and DNAd in mussels. Based on these findings, imatinib could disrupt the repair of oxidized DNAd, leading to the accumulation of alkali-labile sites in DNA. While the short-term exposures resulted in low cytostatic concentrations in mussel tissues, chronic exposure from continuous wastewater releases with the rising use of cytostatic drugs for cancer treatments could still pose a potential environmental risk that warrants further investigation.

**Supplementary Information:**

The online version contains supplementary material available at 10.1007/s10646-025-02976-8.

## Introduction

Over tens of thousands of organic contaminants are released in surface waters from wastewater effluents and urban and industrial runoff. Pharmaceuticals are massively consumed by humans and domestic animals [1]. It is estimated that 10–20% of the parent drugs are excreted in the urine finding their way in municipal effluents [2]. Wastewater treatment plants (WWTP) are ill equipped to remove these compounds that are then delivered into nearby surface waters [3]. It is generally agreed that the excretion of drugs is the main source of environmental contamination of pharmaceuticals [1, 3, 4]. WWTP are not yet equipped to effectively and readily remove polar contaminants which includes multiple pharmaceutical compounds and their metabolites, for example carbamazepine and venlafaxine [5–7]. Many pharmaceuticals are continuously introduced into the environment because they are used to treat chronic diseases or recurrently consumed by the population [1]. Even when these compounds degrade or dissipate, their transformation or removal rates are balanced by this ongoing input. This leads to the pseudo-persistence of these compounds in aquatic environments [8]. This continuous entry of pharmaceuticals as well as their potential active metabolites into the environment can lead to concentrations high enough to induce negative and sublethal effects on aquatic organisms [8]. Antineoplastic drugs in particular can lead to mutagenic, carcinogenic, teratogenic, cytotoxic and genotoxic effects due to their cytotoxic or cytostatic nature even in small concentrations [9, 10]. Aquatic ecosystems that receive wastewater effluents are likely exposed to those substances, and little is known about their effects to aquatic life. Moreover, aquatic organisms are less efficient at biotransforming xenobiotics compared to mammals, because of lower metabolic activity and lack of some cytochrome P450 isoforms involved in drug metabolism [11]. Most drugs are metabolized by CYP1A2, 2C9, 2C19, 2D6 and 3A4 and conjugated by glutathione S-transferase, sulfotransferase and glucuronyl-transferase in mammals [12]. However, the CYP2C family are lacking in fish and mussels while CYP1A and 3 A are present [13]. Hence, these organisms are more susceptible to accumulate pharmaceuticals, perhaps at toxic levels.

Cyclophosphamide (CPA) is an anticancer drug nitrogen mustard and alkylating agent typically used in the treatment of breast, ovarian, and lung cancers and as an immunosuppressant [9]. CPA is eliminated into various human metabolites; carboxyphosphamide being the main human metabolite,^9^ while 4-hydroxycyclophosphamide, 4-ketophosphamide and aldophosphamide are less predominant [14, 15]. CPA and several of its human metabolites can remain unchanged following wastewater treatment with removal under 20%.^16^ The metabolites of CPA also largely retain the genotoxicity and mutagenicity of their parent compound [9, 17]. CPA has been found in hospital and municipal wastewaters in the ngL^− 1^ to µgL^− 1^ range and in surface waters in the pgL^− 1^ to ngL^− 1^ range [9, 18]. The bioaccumulation potential of CPA has been measured empirically in the marine mussel *Mytilus galloprovincialis* at different temperatures and concentrations, though clear trends could not be drawn [19]. CPA bioaccumulation has not yet been assessed in freshwater mussels.

Imatinib (IMT) is a tyrosine kinase inhibitor used in the treatment of leukemia [20]. It is eliminated into various metabolites, N-Desmethyl Imatinib being the predominant one, while IMT itself stays unchanged at 25% of the dose through urine and feces [21]. IMT is resistant to photolysis, hydrolysis and biodegradation [20–22]. Past risk assessment studies have determined that IMT has been found at concentrations over 500 ngL^− 1^ in municipal wastewaters and hospital effluents where concerning adverse effects could be observed [23]. In a recent study, the photocatalytic degradation of IMT using advanced oxidation processes generated transformation products that suggested in silico toxicity comparable or superior to IMT [24]. Imatinib also exhibits estrogenic effect and carries the biggest risk compared to other anticancer drug despite having limited bioaccumulation potential according to in silico models [18, 25]. However, the bioaccumulation potential of IMT has yet to be empirically assessed in aquatic species at environmental concentrations.

In a previous study, freshwater mussels *Elliptio complanata* were exposed to the cytostatic drugs methotrexate and 5-fluorouracil and effects were observed at the biochemical level in mussels exposed to both compounds at environmentally relevant concentration range. Both compounds were taken up in *Elliptio complanata* mussels, but there was no indication of notable bioaccumulation (a few ng g^− 1^ only) after a 96 h exposure. 5-fluorouracil was able to increase dehydrofolate reductase activity, to decrease DNA strand breaks and lipid peroxidation levels in mussel’s gonad tissues. Methotrexate, on the other hand, only significantly increased dehydrofolate reductase activity in the gonad but was significantly correlated with glutathione S-transferase activity [5].

The objective of this study was to further investigate the impacts of cytostatic drugs such as the common CPA and IMT. As such, the mussels *Elliptio complanata* were exposed to increasing concentrations of both compounds for 96 h, then bioaccumulation potential was assessed by a method developed using tandem mass spectrometry coupled with liquid chromatography and toxicological effects were determined with an array of biomarkers of toxicity.

## Materials and methods

### Reagents and standards

Water, acetonitrile (ACN), methanol (MeOH), and formic acid were obtained from Fisher Scientific (Waltham, MA, USA). MiliQ water was obtained from an Advantage A-10 from EMD-MilliPore. Cyclophosphamide-monohydrate (CPA), cyclophosphamide-d4 (CPA-d4), carboxyphosphamide (CPACOOH) and carboxyphosphamide-d4 (CPACOOH-d4) were acquired from Toronto Research Chemical. Imatinib mesylate was acquired from European Pharmacopea Reference Standard and imatinib-d3 was acquired from CND isotopes. As antineoplastics, CPA and IMT were handled with the care and safety measures required. Reference standards in powder form were weighted under fume hood with gloves and protection glasses.

**Table 1:** Physico-chemical properties of the drugs

Drug	CAS/MW/LogP	Predicted BCF^1^/ Measured	Therapeutic mode of action
Cyclophosphamide	6055-19-2/ 261.08/ 0.5	1–10 / 0.28	Cyclophosphamide is an antineoplastic in the class of alkylating agents and is used to treat various forms of cancer. Alkylating agents are so named because of their ability to add alkyl groups to many electronegative groups under conditions present in cells (guanine).
Tyrosine kinase inhibitors (Imatinib)	152459-95-5/ 493.61/ 2.2-5	3–31 / 3	Imatinib is a 2-phenylaminopyrimidine derivative neoplastic agent that belongs to the class of tyrosine kinase inhibitors. Although imatinib inhibits a number of tyrosine kinases, it is quite selective toward the fusion protein that is present in various cancers (apoptosis pathway).

1. From Dimitrov et al., 2002  [37﻿]

### Exposure experiments

Adult freshwater mussels *Elliptio complanata* were collected (>5 cm shell length), under a provincial permit of wildlife services, at pristine lakes devoid of any human influence in the Laurentians area in Québec, Canada. The mussels were collected by hand and shipped back to the laboratory in coolers. They were maintained in 60 L aquarium in dechlorinated tap water and placed on sand bed to allow settling of mussels. They were fed with commercial algal preparation – a mix of 3 commercial phytoplanktons (PhytoGreen-M/ PhytoGreen-S/ PhytoGold, Brightwell Aquatics, USA) – and the water was changed every 2 days. Following 3 weeks acclimation at 15 °C under constant aeration, mussels (10) were placed in 20 L containers lined with polyethylene bags and exposed to increasing concentrations (0, 4, 20 and 100 µgL^− 1^) of cyclophosphamide (CPA) and Imatinib (IMT) for 96 h at 15 °C. Concentrations were selected based on cyclophosphamide levels reported in hospital and wastewater treatment plant (WWTP) influents, as well as on previous experiments with exposed *Mytilus edulis* and *Mytilus galloprovincialis* [19, 26]. Accordingly, a concentration range of 4 to 100 µgL^− 1^ was selected, following a semi-logarithmic scale commonly used in ecotoxicological testing. This range is also sufficiently high to capture potential toxicological effects over a 96-hour exposure period near effluent discharges. The same range was applied to Imatinib to ensure comparability across treatments.

Mussels were not fed during the exposure period. The exposure experiments were repeated 3 times. At the end of the exposure period, mussels were placed in clean aquarium water for 12 h to allow depuration and removal of loosely bound drugs. Mussels were weighted and shell length determined (condition factor-CF) and placed on ice. A 12-hour depuration period was implemented prior to sampling to assess the bioaccumulation and biological effects of internalized contaminants while minimizing interference from loosely adsorbed substances. This approach is consistent with OECD Test Guideline 305, which provides standardized procedures for evaluating bioaccumulation in aquatic organisms. Supporting evidence from mussel studies confirms that short depuration does not eliminate biologically relevant data [27, 28]. Moreover, *Dreissena polymorpha* exposed to benzo[a]pyrene retained DNA adducts and showed sustained expression of detoxification genes (AHR, GST, CAT) even after 28 days of depuration [29].

### Sampling and extraction of antineoplastic drugs

The protocols for the extraction of the antineoplastic drugs as well as the method validations are shown in the Supplementary Information and Table **S1**.

### Chromatography and mass spectrometry

A Thermo Scientific TSQ Altis plus triple quadrupole mass spectrometer (San Jose, CA, USA) was interfaced with a Thermo Scientific UHPLC system using a pneumatic assisted heated electrospray ion source. A 2.1 × 100 mm BEH C18 column with 1.7 μm particle size was used for chromatographic separation. The details of the liquid chromatography method, the source parameters of the mass spectrometer and the details of the multiple reaction monitoring method are shown in the Supplementary Information in Sect. 1.2, Fig **S1**, Fig S2 while the limits of detection and quantification of the method for the antineoplastic dugs are shown in Table S2.

### Biomarkers analyses

The digestive gland and gonad tissues were removed, immersed in ice-cold homogenization buffer (25 mM Hepes–NaOH, pH 7.4, 100 mM NaCl, 0.1 mM dithiothreitol, and 1 µgL^− 1^ of aprotinin) and homogenized using a Polytron tissue grinder at 4 °C (2 × 30 s burst at mid power to prevent excessive heating). A portion of the homogenate was kept aside for lipid peroxidation (LPO) and DNA damage (DNAd) assessments. The remainder of the homogenate was centrifuged at 12 000 x g for 20 min at 2 °C and the supernatant (S12 fraction) was collected for arachidonate cyclooxygenase (COX; inflammation marker), glutathione S-transferase (GST; phase II biotransformation of drugs) and acetylcholinesterase (AChE; neural activity) activities.

Total proteins were determined in the homogenization and S12 fraction to normalize the above endpoints [30]. LPO levels were evaluated using the thiobarbituric acid reactants for malonaldehyde [31]. Calibration was achieved using tetramethoxypropane solutions and fluorescence readings were taken at 540-nm excitation and 600-nm emission in 96-well dark microplates (Synergy-IV, Bioteck Instruments, CA, USA). Data were expressed as nmoles of TBARS per mg of homogenate proteins. The levels in DNAd were determined using the alkaline precipitation assay (APA) using fluorescence determination of DNA strands [32, 33]. The measurement of DNA strands remaining in the supernatants was achieved with the Hoechst dye at 350-nm excitation and 450-nm emission in 0.3 M NaCl, 0.2 M Tris base, and 4 mM sodium cholate to minimize interferences from traces of SDS and pH [34]. Data were expressed as µg DNA × mg^− 1^ proteins in the homogenate. GST activity was performed by the method described by Gagné using 1 mM of 1-chloro-2.4-dichloronitrobenzene (CDNB) as substrate and 1 mM reduced gluthathione in Hepes–NaOH, pH 6.8 [35]. The absorbance was measured at 340 nm. and the results were expressed as increased absorbance/minute/mg protein. For the COX activity, the assay measured the formation of H_2_O_2_ from the oxidation of arachidonic acid as previously described [36]. Fluorescein from the 2,4-dichlorofluorescein oxidation was measured at 485 nm for excitation and 520 nm for emission using a microplate reader (Synergy 4, BioTek, USA). The data was expressed as the rate of increase in relative fluorescence units (RFU)/ (min × mg proteins).

### Statistical analysis

Normality and homogeneity of variances of the biomarker data were tested using the Shapiro–Wilk and Levene’s tests, respectively. Either one-way ANOVA with Tukey’s post-hoc test or non-parametric ANOVA with the Conover–Iman test was applied. Pearson’s correlation analysis was performed to explore relationships among biomarkers. To identify patterns in biomarker responses and group similarities, hierarchical cluster analysis was conducted. Additionally, discriminant function analysis was used to determine the similarities between the bioaccumulation of the respective drugs and biomarker data. All statistical analyses were performed using the SYSTAT software package (version 13, San Jose, CA, USA), and significance was set at *p* < 0.05.

## Results and Discussion

### Cyclophosphamide

**Fig. 1:**
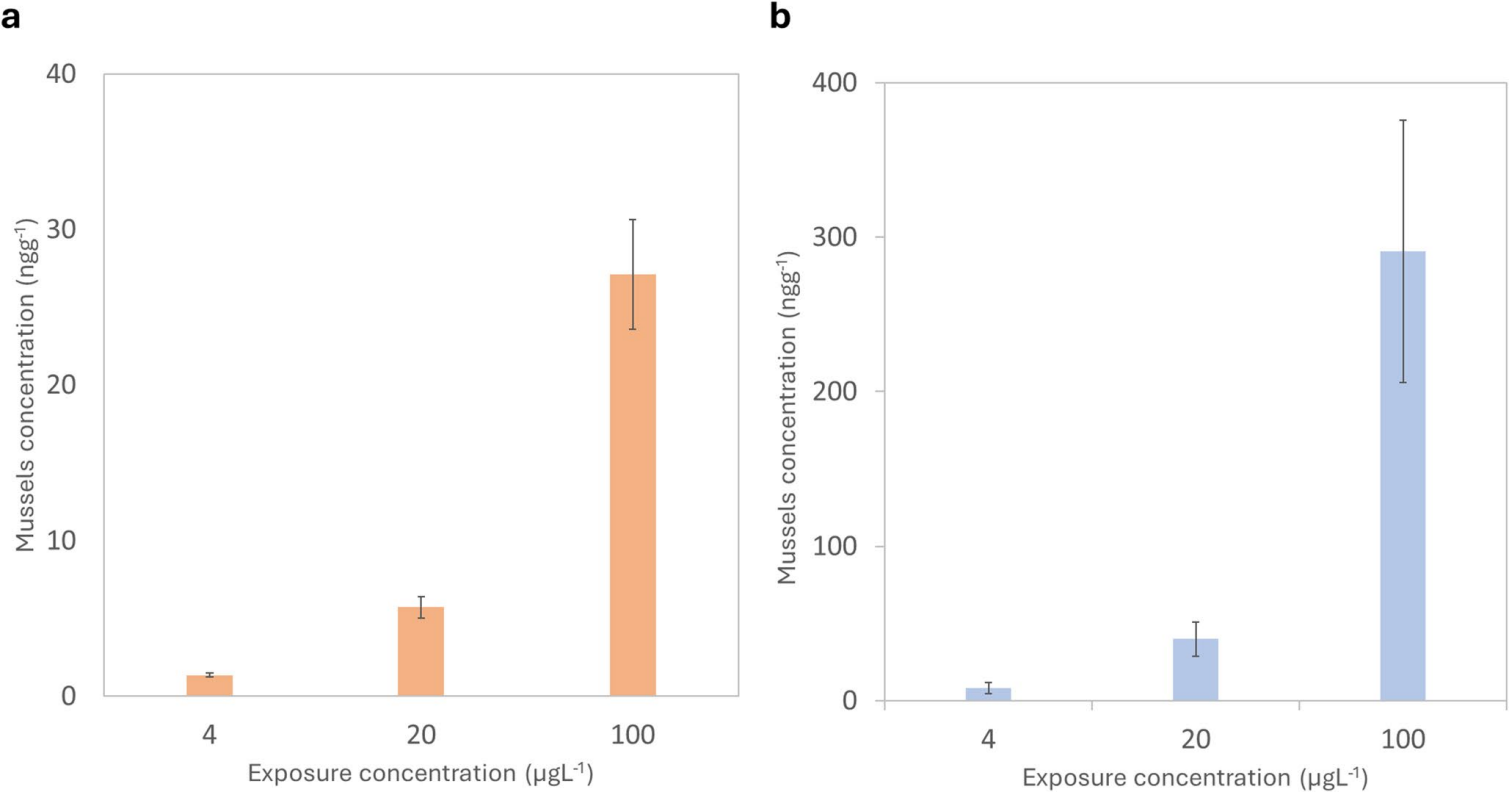
Average concentration of **(a)** cyclophosphamide and **(b)** Imatinib in *Elliptio complanata* mussels (ngg^-1^) per exposure concentration (µgL^-1^) (*n* = 10 mussels per exposure concentration, each mussel analyzed in triplicate)

**Fig. 2:**
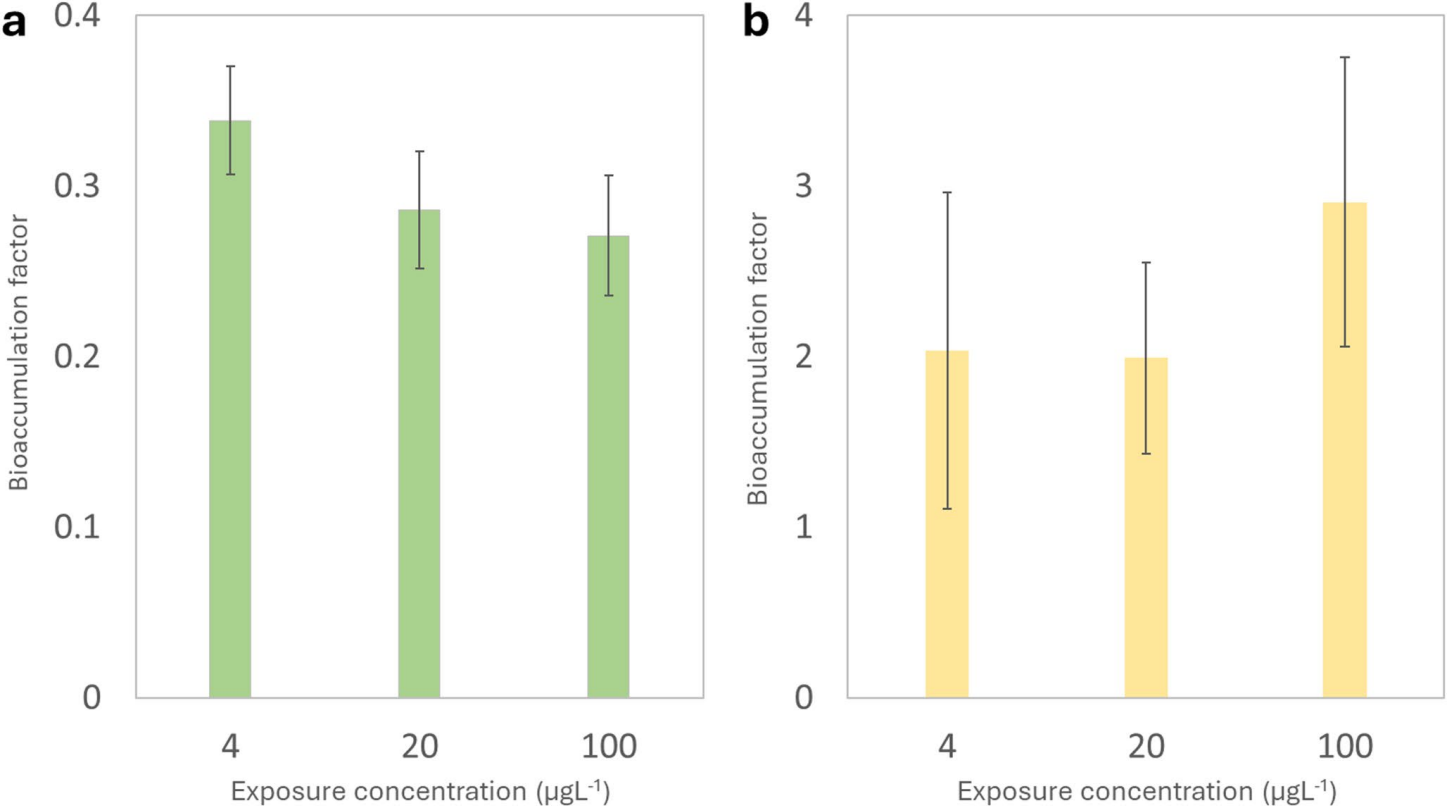
Bioaccumulation factor (BCF) for **(a)** CPA and **(b)** IMT per exposure concentration (µgL^− 1^). BCF is expressed as the average concentration in Elliptio complanata mussels divided by the exposure concentration. (*n* = 10 mussels per exposure concentration, each mussel analyzed in triplicate)

This study documents the bioaccumulation of CPA in tissues of bivalves and the first to do examination for carboxy-CPA. Mussels were exposed under controlled conditions to different concentrations (4–100 µgL^− 1^) of cyclophosphamide (CPA) and measured concentrations were observed comparable to the theoretical/nominal concentrations of the exposition media (Table S3). Significant increases in CPA tissue concentrations were observed in exposed mussels (Fig. 1a). Up to 27 ngg^− 1^ was taken up for a 72 h- exposure to 100 µgL^− 1^. When expressed relative to exposure concentration (i.e., bioaccumulation factor, BCF), CPA exhibited low bioaccumulation potential (Fig. 2a), giving a BCF of 0.27. This value was below the predicted BCF value (1–10) using the in silico method for CPA (Table 1**)**. This could suggest that the main driver of accumulation was not entirely dependent on lipophilicity, but rather on the pKa (ionization) or molecular size [37]. CPA is a relatively small molecule, but the two ionizable amine groups indicating that size effects were minimal for this compound. When expressed relative to exposure concentration (i.e., bioaccumulation factor, BAF), CPA exhibited low bioaccumulation potential (Fig. 2a). The metabolite carboxyphosphamide, transformation product of accumulated CPA, was tentatively investigated and levels in all mussel tissue samples were below the limit of detection (< 0.11 ngg^− 1^) (Table S2). This could suggest that mussels do not biotransform this pharmaceutical or that it was degraded prior to analysis. It appears that CPA is mainly metabolized by cytochrome P4502C isoform in keeping with the notion that most pharmaceuticals are metabolized by the following isoforms in mammals: 1A2, 2C9, 2C19, 2D6 and 3A4 [12, 38]. This is consistent to a previously reported study that mussels have lower cytochrome P450 activity and lacked the 2 C family [13].

**Fig. 3:**
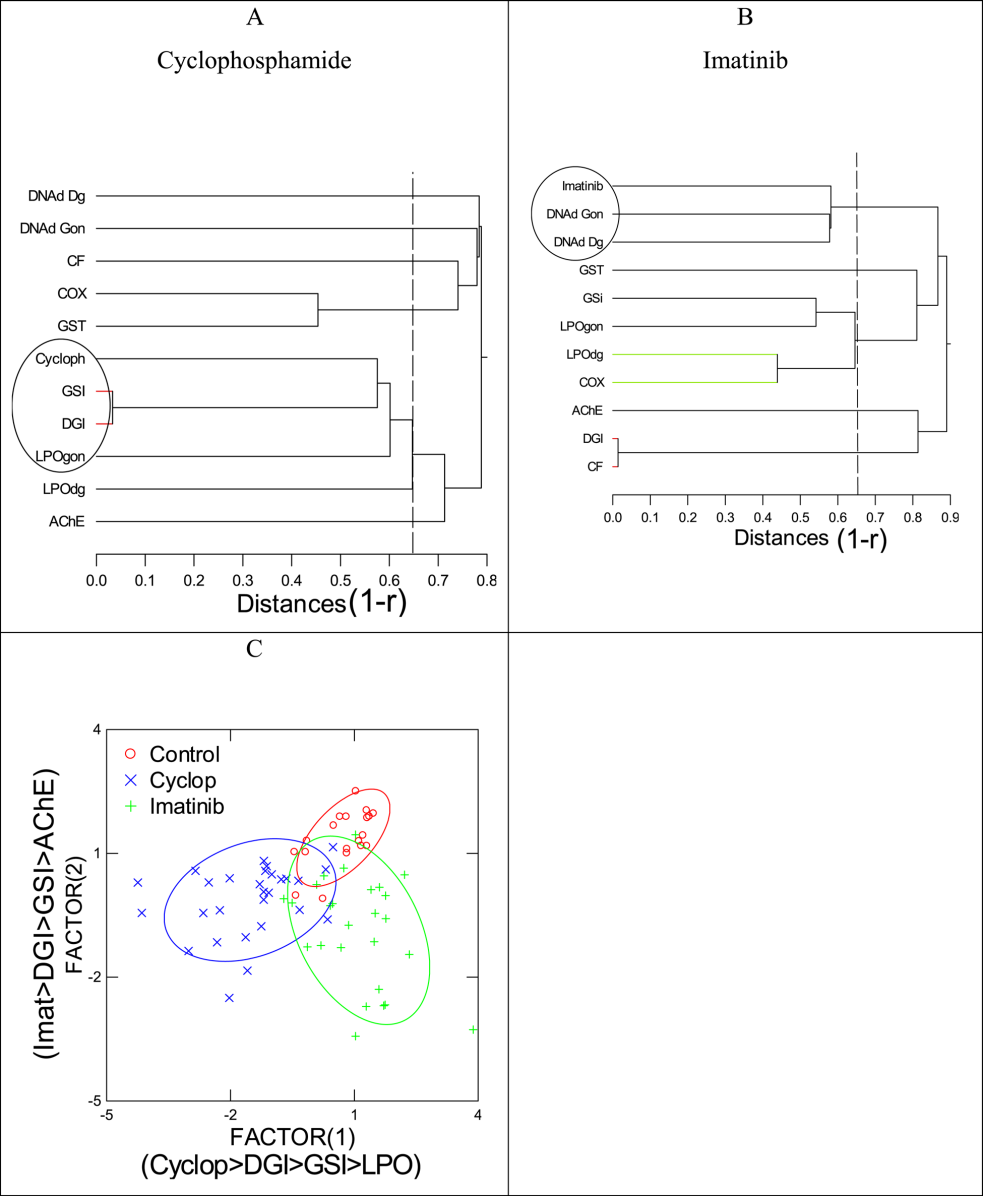
Hierarchical tree and discriminant analyses of biomarker data. Hierarchical tree of biomarker data for cyclophosphamide (**A**) and imatinib (**B**) are shown. The dotted lines correspond to the significance threshold (p < 0.05) of the distance metric 1-r. Discriminant function analysis was performed to determine the similarities between the bioaccumulation of the respective drugs and biomarker data (**C**). All the variance was explained with a classification accuracy of 73%. The major biomarkers (showing the highest correlation with each factor) were drug levels in tissues, DGI, GSI, LPO in gonads and AChE

In the attempt to better understand the toxic mode of action of CPA, a suite of biomarkers was undertaken in the digestive gland and gonad tissues in mussels (Table 2). The data revealed that CPA exposures significantly reduced AChE, GST and COX activities at 4 µgL^− 1^ and DNA strand breaks at 20 µgL^− 1^ in the gonad. The drug also increased the glutathione S-transferase activity (GSI) and the oxidative stress (LPO) in both the digestive gland and gonad. Hierarchical tree analysis with the correlation coefficient (1-r) as the distance metric revealed that tissue levels of CPA were significantly correlated with LPO in gonads, DGI and GSI (Fig. 3) which could suggest that it is involved in oxidative stress (LPO) and increased GSI.

**Table 2:** Biomarker response data for mussels exposed to cyclophosphamide (CPA) and Imatinib (IMT)

Exposure concentration (ugL^− 1^)	CF CPA IMT	DGI CPA IMT	GSI CPA IMT	AChE CPA IMT	GST CPA IMT	COX CPA IMT	DG LPO CPA IMT	Gon LPO CPA IMT	DG DNAd CPA IMT	Gon DNAd CPA IMT
0	0.6 − 0.02 0.49 − 0.01	0.018 − 0.002 0.017 − 0.003	0.032 − 0.003 0.034 − 0.002	0.057 − 0.006 0.065 − 0.005	0.39 − 0.02 0.38 − 0.01	270 − 15 272 − 30	2.1–0.2 1.9 − 0.1	2-0.3 1.8 − 0.1	0.40 − 0.04 0.38 − 0.05	0.46 − 0.08 0.52 − 0.1
4	0.57 − 0.03 0.50 − 0.01	0.015 − 0.001 0.017 − 0.002	0.031 − 0.002 0.04 − 0.002*	0.05 − 0.006* 0.05 − 0.007*	0.30 − 0.02* 0.34 − 0.02*	230 − 11* 261 − 17	2-0.3 2-0.1	2-0.3 4-0.4*	0.45 − 0.03 0.39 − 0.05	0.35 − 0.06 0.38 − 0.1*
20	0.59 − 0.04 0.50 − 0.01	0.017 − 0.002 0.017 − 0.005	0.04 − 0.01* 0.04 − 0.004*	0.044 − 0.008* 0.065 − 0.005	0.32 − 0.02* 0.36 − 0.01	215 − 11* 240 − 21	3-0.1* 3 − 0.l	3-0.1* 4-0.1*	0.35 − 0.03 0.37 − 0.02	0.27 − 0.07* 0.21 − 0.01*
100	0.54 − 0.07 0.49 − 0.01	0.03 − 0.015 0.02 − 0.001	0.08 − 0.04* 0.039 − 0.03	0.05 − 0.005 0.04 − 0.008*	0.38 − 0.01 0.36 − 0.02	231 − 10* 231 − 20	3-0.2* 2-0.1	3-0.1* 3-0.1*	0.46 − 0.03 0.48 − 0.04*	0.2-0.008* 1.1–0.3*

CF: Condition factor. DGI: Digestive gland index. GSI: Gonadosomatic index. AChE: Acetyl Choline Esterase Activity. GST: Glutathione S-transferase Activity. COX: Cyclooxigenase Activity. DG LPO : Digestive gland lipid peroxidase assay. Gon LPO: Gonade lipid peroxidase assay. DG DNAd: Digestive gland DNA damage. Gon DNAd: Gonad DNA damage

Although the digestive gland index (DGI) was also correlated with these parameters, no significant change with the exposure concentration was observed. The “gonadotoxicity” of CPA was previously reported in rats [39]. An increase in GSI was observed in rats exposed to CPA, similar to the increase seen in the mussel study. CPA induces oxidative stress, inflammation, and fibrosis activities in the gonads of adult male rats, which would be consistent with the effects observed on LPO in mussels in this study. Moreover, gonad DNA strand breaks were significantly reduced by CPA potentially suggesting decreased DNA metabolism and turnover (repair activity). CPA is an alkylating agent known to alkylate DNA forming interstrand crosslinks, which can decrease DNA strand levels by the alkaline precipitation methodology [40]. The repair of alkylated DNA intercross links required a transcription-couples nucleotide excision repair mechanisms, which can be readily increased by CPA [41]. However, it should be noted that no evidence of such mechanisms was observed in mussels, as no changes in DNA strand breaks were observed.

### Imatinib

In contrast to CPA, gradually lower measured concentrations of IMT in exposure media were observed compared to the theoretical concentrations of the exposition media (Table S3). The concentration of the new solution for the 4 µgL^− 1^ solution was of 2.4 ± 0.1, which is 60% and the concentration of the 100 µgL^− 1^ solution was 91.8 ± 0.7 at the end of the exposure. This could be explained by the adsorption of IMT to the surface of the aquarium during the exposition or the degradation of the compound.

The concentrations of IMT in *Elliptio complanata* mussels suggest a stronger bioaccumulation potential than for CPA as shown in Fig. 2b; the measured uptake of IMT was one order of magnitude higher than that of CPA. Concentrations in tissues as high as 300 ngg^− 1^ were observed after a 96 h- exposure period, giving a BCF of 3 (Fig. 3b). As a result, accumulation in mussels expressed as BCF was higher but not significant among the increasing exposure concentrations. The measured BCF value (3) was in the same range of the predicted BCF based on octanol-water coefficient shown in Table 1 [37]. This could suggest that lipophilicity, not pKa, was the main driver of accumulation in mussels. Much less accumulation was reported for the cytostatic methotrexate in mussels exposed to similar concentrations with bioaccumulation concentrations lower than 3 ngg^− 1^.^2^ Such results pointed out the low bioaccumulation potential (i.e., BAF < 3) despite CPA and IMT being bioaccumulated by mussels. This could indicate a saturation/regulation in mussel tissues with increased concentrations of cytostatics [2]. Imatinib was shown to undergo cytochrome P450 biotransformation with the following isoforms: CYP1A1, CYP1B1, CYP3A4 and FMO3 [42]. CYP3A4 activity was detected in various invertebrates, which could suggest that this compound can be metabolized, in part at least, in mussels and fish [11].

In the attempt to better understand the toxic mode of action of IMT, a suite of biomarkers was undertaken in the digestive gland and gonad tissues in mussels (Table 2). The following biomarkers were increased in mussels exposed to IMT: GSI, gonad LPO, DNAd in digestive gland and gonad. AChE activity was decreased in mussels exposed to IMT. Hierarchical tree analysis with the correlation coefficient (1-r) as the distance metric revealed that tissue levels of IMT was significantly correlated with DNAd in both the digestive gland and gonads (Fig. 3b). The decreased in AChE activity was also involved in the main effects of IMT based on discriminant function analysis (Fig. 3c). This would suggest that IMT is involved in decreased neural activity and DNA damage in mussels. The formation of DNA strand breaks is thought to arise from inhibition of pyrimidine tyrosine kinase targeting BCR/ABL oncogene [43]. According to this study, IMT did not induce DNA strand breaks from direct interaction to DNA. The formation of DNA strand breaks resulted from the formation of alkali-labile sites rather than from strand break formation [43]. In the presence of IMT, K562 cells were unable to repair H_2_O_2_-induced DNA damage if they were pre-incubated with IMT (ST1571) [43]. This could suggest that IMT could disrupt the repair of oxidized DNA damage, leading to the accumulation of alkali-labile sites in DNA. This could further imply that free radical formation (which occurs during the normal metabolic/respiration in cells) is involved in the formation of DNA lesions induced by IMT.

Given that the predictive environmental concentration of Imatinib is in the order of 4.2 µgL^− 1^ but could reach 26 ugL^− 1^ when we consider all drugs with the same mode of action – i.e. tyrosine kinase inhibitors – this concentration would then potentially be within the exposure concentration used in this study [44]. This would then suggest that tyrosine kinase inhibitors could lead to harmful effects in freshwater mussels near municipal effluent discharges in these circumstances. This conclusion would concur with the recent observation that municipal effluents are genotoxic to mussels [45, 46]. Indeed, a recent survey of municipal effluents revealed that 60% of them were potentially genotoxic based on an enzyme-based DNA protection index [47].

However, additional studies with lower exposure concentrations would provide more information on the effect of IMT farther away from municipal effluent discharges. Additionally, more studies documenting the concentration of IMT and other tyrosine kinase inhibitors in wastewater effluents and surface waters would allow to better evaluate the risk. More information is also needed on the effects of other highly used antineoplastic drugs. In particular, the impacts of doxorubicin on aqueous species need to be evaluated as it is a widely used antineoplastic drug with very little information available.

## Conclusion

The results suggest that short-term exposure of mussels to CPA and IMT leads to relatively low cytostatic concentration in tissue, but chronic exposure due to constant wastewater releases is still a potential environmental risk that needs further investigations given mussels lack some of the drug metabolizing enzymes. IMT was more bioavailable in mussels compared to CPA. Biomarker analyses indicated that CPA and IMT displayed different modes of action in mussels. CPA increased GSI and LPO in both the digestive gland and gonad in mussels while IMT led to DNA strand breaks in the digestive gland and gonad. These effects are consistent with the therapeutic mode of action of CPA and IMT. Based on the predicted environmental concentrations of tyrosine kinase inhibitors, their levels could reach concentrations in tissues able to potentially elicit toxic effects in freshwater mussels in keeping with the genotoxic potential of municipal wastewaters. Given the increasing use of cytostatic drugs by the human population against cancers, their levels found in effluents and receiving waters should be monitored to protect local and sessile residents such as freshwater mussels [48].

## Supplementary Information

Below is the link to the electronic supplementary material.

Supplementary Material 1

## Data Availability

No datasets were generated or analysed during the current study.
