# Supplementary material for: Bioaccumulation and toxicity of imatinib, cyclophosphamide and its transformation product carboxyphosphamide in the freshwater mussel Elliptio complanata
Source: Ecotoxicology. 2025 Oct 10;34(10):2112–21. doi: 10.1007/s10646-025-02976-8 (PMC12619791; doi:10.1007/s10646-025-02976-8)
Supplement: Supplementary file 1 — Supplementary Material 1 [file 10646_2025_2976_MOESM1_ESM.docx]

Bioaccumulation and toxicity of imatinib, cyclophosphamide and its transformation product carboxyphosphamide in the freshwater mussel *Elliptio complanata*

Eysseric E, Vlassopoulos L, André C, Gagné F, Gagnon C.

Environment and Climate Change Canada, Aquatic Contaminant Research Divison, 105 McGill, Montreal, Qc, Canada. H2Y 2E7.

Supplementary Information

# Materials and methods

## Sampling and extraction of antineoplastic drugs

### Cyclophosphamide

The digestive glands, gonads, and gills were harvested and immediately frozen at -80C. Upon extraction and analysis, the mussels tissues were thawed and homogenized with a polytron BioHomogenizer from Biospec products after which the homogenates were subdivided into three 500 mg replicates in 15 mL conical centrifuge polypropylene tubes. Then, 1.5 mL of ACN, 1.5 H2O with 100 mM Na4CO3 with pH adjusted at 9.5 were added following 40 µL of each 100 ppb internal standards solutions of CPA-d4 and CPACOOH-d4. The samples were vortexed for 30 seconds and centrifuged at 3000g for 30 minutes. The supernatant was transferred into a 50 mL polypropylene conical centrifuge tube and miliQ water was added at up to the 50 mL mark. Then, the samples were extracted with a 150 mg (6 cc) Oasis MAX cartridge (Waters) priorly conditioned with 6 mL methanol and equilibrated with 6 mL H2O. The cartridges were washed with 6 mL H2O and frozen upon analysis.

Cartridges were eluted two times with 3 mL of a solution of ACN : MeOH 1 : 1 (V/V) with 5% formic acid. The samples were dried under nitrogen flow and reconstituted with 70% H2O and 30% MeOH : ACN 3:1 (V/V), namely the initial liquid chromatography conditions. Recoveries for CPA are shown in **Table S1**.

Table S1 Recoveries of the compounds in the methods

| **Compound** | **Recovery (%)** |
| --- | --- |
| Cyclophosphamide | 52 |
| Carboxyphosphamide | 30 |
| Imatinib | 80 |

As the exposure aquarium water was changed every 24 hours, the concentrations of the drugs at T0 and T24h were also determined. For the extraction, 1.5 mL of the exposure water was mixed with milli-Q to 10 mL and loaded in a 150 mg (6 cc) Oasis MAX cartridge (Waters) priorly conditioned with 6 mL methanol and equilibrated with 6 mL H2O. The cartridges were washed with 6 mL H2O and frozen upon analysis.

### Imatinib

The same procedure as for the cyclophosphamide homogenization was applied for imatinib. For the extraction, 1.5 mL of ACN, 1.5 of H2O with 5% NH4OH, and 40 µL of a 100 ppb internal standard solution of IMT-d3 was used. The samples were vortexed for 30 seconds and centrifuged at 3000g for 30 minutes. The supernatant was transferred into a 50 mL polypropylene conical centrifuge tube and milliQ water was added at up to the 50 mL mark. Then, the samples were extracted with a 200 mg (6 cc) Oasis HLB cartridge (Waters) priorly conditioned with 6 mL methanol and equilibrated with 6 mL H2O. The cartridges were washed with 6 mL H2O and frozen upon analysis. Cartridges were eluted with two times 3 mL of a solution of ACN : MeOH 1 : 1 (V/V). The samples were dried under nitrogen flow and reconstituted with 70% H2O and 30% MeOH : ACN 3:1 (V/V). Recoveries for IMT are shown in **Table S1**.

Similar procedure for the renewal of the exposure media was used for IMT. For the extraction, 1.5 mL of the exposure water was diluted with milli-Q to 10 mL then loaded in a 200 mg 6 cc Oasis HLB cartridge (Waters) priorly conditioned with 6 mL methanol and equilibrated with 6 mL H2O. The cartridges were washed with 6 mL H2O and frozen upon analysis.

## Chromatography and detection methods

### Cyclophosphamide

The Mobile mobile phase A was consisted of H2O with 0.2% formic acid (V/V or v/v-minuscule) and mobile phase B was MeOH : ACN 3:1 (V/V) with 0.2% formic acid (V/V). The LC gradient was as follow: at initial time, 30% B; at 2 minutes, 100% B, at 3 minutes, 100%; at 3.01 minutes, 30% B; at 5 minutes 30% B. The method elution time was took 5 minutes long. The LC gradient is shown in **Fig. S1**. The retention times were 2.31 min for CPA and CPA-d4 and 1.74 min for CPACOOH and CPACOOH-d4.

Fig. S1 Liquid-Chromatography gradient for the analysis of cyclophosphamide by percentage of mobile phase B (MeOH : ACN 3:1 (V/V) with 0.2% formic acid (V/V))

For the MS method, the precursor ions for CPA, CPA-d4, CPACOOH and CPACOOH-d4 were 261, 267, 293, and 299 respectively and the product ions were 120, 142, 221, and 227 respectively. Collision energies were 27V, 24V, 19V, and 19V for CPA, CPA-d4, CPACOOH, CPACOOH-d4. RF lens voltages were 71V, 71V, 58V, and 57V for CPA, CPA-d4, CPACOOH, CPACOOH-d4. Spray voltage was 3900, sheath gas 50, auxiliary gas was 10, sweep gas was 1, ion transfer tube temperature was 325°C, vaporizer was 350°C, dwell time was 97ms. Limits of detection and quantification of the method for CPA are shown in **Table S2**.

Table S2 Limits of detection and quantification of the methods

| **Compound** | **Limit of detection (ng/g)** | **Limit of quantification (ng/g)** |
| --- | --- | --- |
| Cyclophosphamide | 0.08 | 0.3 |
| Carboxyphosphamide | 0.11 | 0.4 |
| Imatinib | 0.47 | 1.6 |

### Imatinib

For imatinib, mobile phase was H_2_O with 5mM ammonium acetate with pH adjusted at 9.25 with NH4OH and mobile phase B was MeOH : ACN 3:1, 5mM ammonium acetate with pH adjusted at 9.25. The LC method gradient was as follow: at initial time, 30% B; at 2.5 minutes, 100% B; at 4 minutes, 100% B; at 4.01 minutes, 30% B; at 6 minutes, 30% B. The method was 6 minutes long. The LC method is shown in **Fig. S2**. The retention time of both IMT and IMT-d3 was 3.03 minutes.

Fig. S2 Liquid-Chromatography gradient for the analysis of imatinib by percentage of mobile phase B (MeOH : ACN 3:1 (V/V) with 0.2% formic acid (V/V))

For the MS method, the precursor ions for IMT and IMT-d3 were 494 and 497 respectively and the product ion was 394 for both compounds. Collision energy was 25V for both compounds, RF lens was 90V, spray voltage was 3850, sheath gas 50, auxiliary gas was 10, sweep gas was 1, ion transfer tube temperature was 325°C, vaporizer was 350°C, dwell time was 97ms.

As methods validation, recoveries of the compounds as well as limits of detection and quantification are reported in **Tables S1 & S2**. The higher limits of detection and quantification for Imatinib were due to higher variability in calibration standards.

# Results & discussion

**Table S3** Concentrations of cyclophosphamide (CYCP) and Imatinib (IMT) in exposure medium

| **Compound** | **Nominal concentration (µgL^-1^)** | **Time of exposure (hours)** | **Measured initial concentration (T_0_) (µgL^-1^)** | **Measured concentration when replaced after 24 hours (T_0_ +24 hours) (µgL^-1^)** |
| --- | --- | --- | --- | --- |
| CYCP | 4 | 0 | 4 | N/A^*^ |
|  |  | 24 | 3.9 ± 0.4 | 3.7 ± 0.4 |
|  |  | 48 | 4.3 ± 0.4 | 3.4 ± 0.3 |
|  |  | 72 | 4.6 ± 0.5 | 4.1 ± 0.4 |
|  | 20 | 0 | 20 | 18 ± 2 |
|  |  | 24 | 18 ± 2 | 14 ± 1 |
|  |  | 48 | 18 ± 2 | 16 ± 2 |
|  |  | 72 | 20 ± 2 | 18 ± 2 |
|  | 100 | 0 | 100 | 88 ± 8 |
|  |  | 24 | 93 ± 8 | 81 ± 7 |
|  |  | 48 | 96 ± 9 | 91 ± 8 |
|  |  | 72 | 85 ± 8 | N/A^*^ |
| IMT | 4 | 0 | 4 | N/A^*^ |
|  |  | 24 | N/A^*^ | N/A^*^ |
|  |  | 48 | N/A^*^ | 1.4 ± 0.4 |
|  |  | 72 | 2.4 ± 0.1 | 1.4 ± 0.3 |
|  | 20 | 0 | 20 | N/A^*^ |
|  |  | 24 | N/A^*^ | N/A^*^ |
|  |  | 48 | N/A^*^ | 8.0 ± 0.1 |
|  |  | 72 | 14.1 ± 0.2 | 8.5 ± 0.4 |
|  | 100 | 0 | 100 | N/A^*^ |
|  |  | 24 | N/A^*^ | N/A^*^ |
|  |  | 48 | N/A^*^ | 76 ± 4 |
|  |  | 72 | 91.8 ± 0.7 | 74.1 ± 0.2 |

*: Not available
